# Molecular testing, first-line treatment patterns, and survival in metastatic Colombian non–small cell lung cancer: the RECAPC multicenter registry

**DOI:** 10.3389/fonc.2026.1863940

**Published:** 2026-07-17

**Authors:** Ricardo Brugés, Pedro Ramos, Milton Lombana, Anderson Osma, Néstor Llinás, Javier Cuello, Andrés Yepes, Ray Manneh, Anabelly Coronel, Rebeca Granadillo, Carolina López, Álvaro Osorio, Daniel Santa, Natalia Arango, William Mantilla, Diego Gómez

**Affiliations:** 1 Instituto Nacional de Cancerología, Empresa Social del Estado (ESE), Bogotá, Colombia; 2 Pontificia Universidad Javeriana, Bogotá, Colombia; 3 Hospital Universitario San Ignacio, Bogotá, Colombia; 4 Clínica Universitaria Colombia (Keralty), Bogotá, Colombia; 5 Clínica de Occidente S.A., Cali, Colombia; 6 Fundación Colombiana de Cancerología Clínica Vida, Medellín, Colombia; 7 Centro Oncológico de Antioquia S.A.S. (COA), Medellín, Colombia; 8 Sociedad de Oncología y Hematología del Cesar S.A.S. (SOHEC), Valledupar, Colombia; 9 IDC Instituto de Cáncer Hemato Oncólogos S.A., Cali, Colombia; 10 Fundación Valle del Lili, Cali, Colombia; 11 Clínica Medellín – Grupo Quirónsalud, Medellín, Colombia; 12 Fundación Cardioinfantil – Instituto de Cardiología, Bogotá, Colombia; 13 Hospital Internacional de Colombia (HIC), Fundación Cardiovascular de Colombia (FCV), Bucaramanga, Colombia

**Keywords:** biomarkers, non-small-cell lung cancer, immunotherapy, molecular targeted therapy, neoplasm metastasis, registries, survival analysis

## Abstract

**Background:**

Molecular profiling and programmed death-ligand 1 (PD-L1) status guide first-line treatment selection in metastatic non–small cell lung cancer, yet real-world testing, treatment delivery, and documentation vary across health systems. We described molecular testing documentation, first-line treatment patterns, safety capture, and survival outcomes in a Colombian multicenter registry cohort.

**Methods:**

We conducted a retrospective registry-based cohort study using the RECAPC central repository. Data extraction was finalized on October 10, 2025 (registry cutoff). The analytic cohort included patients with metastatic-at-diagnosis disease. Driver status was summarized using a prespecified mutually exclusive cascade (EGFR/ALK altered; Other actionable altered; no driver detected; not tested/unknown). First-line treatment was classified into five categories. Overall survival was estimated with Kaplan–Meier methods and modeled using Cox regression. Safety variables were obtained as binary indicators by treatment line.

**Results:**

The cohort included 585 patients; mean age was 72.0 years and 52.0% were female. Driver groups were EGFR/ALK altered (32.8%), Other actionable altered (4.6%), no driver detected (15.0%), and not tested/unknown (47.5%). First-line therapy was chemotherapy alone (34.4%), chemo-immunotherapy (21.0%), immunotherapy alone (3.9%), targeted therapy (26.7%), and not reported (14.0%). PD-L1 expression was <1% in 149 patients (25.5%), 1–49% in 119 (20.3%), and ≥50% in 74 (12.6%); testing was not performed in 156 (26.7%), and data were missing in 87 (14.9%). Among 342 patients with an interpretable PD-L1 result, 193 (56.4%) had PD-L1 ≥1% and 74 (21.6%) had PD-L1 ≥50%. Overall survival was evaluable in 490 patients with 229 deaths; median follow-up was 34.99 months. Survival differed by driver group (log-rank P < 0.001). In adjusted analyses, not tested/unknown remained associated with higher mortality versus EGFR/ALK altered (hazard ratio 2.26; 95% CI, 1.62–3.16; P < 0.001). Age, expressed per 10-year increase, was also associated with mortality (hazard ratio 1.28; 95% CI, 1.10–1.48; P < 0.001), as was ECOG performance status 2 (hazard ratio 1.76; 95% CI, 1.10–2.81; P = 0.02). No PD-L1 category was independently associated with overall survival relative to <1%. A single cutaneous adverse event was recorded in one patient receiving targeted therapy.

**Conclusions:**

In this Colombian registry cohort, incomplete molecular characterization and missing first-line regimen documentation were common. In this setting, missing regimen data likely reflect both incomplete capture and discontinuities across care pathways. Findings should be interpreted as observational associations rather than causal effects.

## Introduction

Metastatic non–small cell lung cancer remains a leading cause of cancer mortality, and outcomes increasingly depend on timely identification of actionable oncogenic drivers and programmed death-ligand 1 expression to guide first-line treatment selection (1). Landmark trials established durable benefit with biomarker-matched therapy, including pembrolizumab monotherapy in patients with high programmed death-ligand 1 expression and no targetable drivers, osimertinib in epidermal growth factor receptor–mutated disease, and contemporary ALK-directed therapy in ALK-rearranged tumors (2–4). In routine practice, biomarker testing and treatment delivery vary across health systems and care settings, with persistent gaps even in well-resourced environments and marked barriers in lower- and middle-income settings (5–9).

Latin America faces additional complexity because the distribution of driver alterations differs from other regions, and access to molecular testing and targeted therapies is heterogeneous across countries and within health systems (10, 11). Regional real-world evidence suggests that broader genomic profiling can identify actionable alterations and support treatment individualization, yet the feasibility of implementing comprehensive testing and delivering matched therapies remains uneven (12, 13). Observational evidence from Latin American collaborations also shows variable adoption of immune checkpoint inhibitors in metastatic disease, shaped by access and practice patterns rather than trial-like selection (14).

Colombia adds a layer of system-level complexity that is directly relevant to interpreting registry-based oncology data. Qualitative work in public healthcare networks has identified insurer-driven authorization processes and fragmented provider contracting as prominent barriers that delay diagnosis and treatment (15). Geographic concentration of oncology services and travel burdens further widen inequities, particularly outside major urban centers (16, 17). In that setting, missing treatment documentation in a multicenter registry can reflect incomplete data transfer across fragmented provider networks, delays in care delivery, or both, rather than a purely clerical omission (18, 19).

RECAPC is an initiative of the Colombian Association of Hematology and Oncology that consolidates multicenter real-world data to describe contemporary cancer care in Colombia. In this registry-based cohort study, we aimed to describe (1) molecular testing documentation and driver grouping, (2) first-line treatment patterns and their temporal adoption, and (3) overall survival and treatment-trajectory outcomes in patients with metastatic-at-diagnosis non–small cell lung cancer treated across participating Colombian centers. The primary analyses were descriptive, and the retrospective survival models were intended to characterize associations rather than support causal inference.

## Methods

### Study design and data source

RECAPC is an initiative of the Colombian Association of Hematology and Oncology (ACHO) that assembles real-world clinical data from participating Colombian institutions. We conducted a multicenter retrospective registry-based cohort study using the RECAPC central repository, integrating baseline clinical characteristics, molecular testing results, systemic therapy lines, and outcomes.

Data extraction from the RECAPC central repository was finalized on October 10, 2025 (registry cutoff). The analytic datasets were subsequently locked for reproducible analysis and stored as hash-verified files. The resulting extract included 585 metastatic-at-diagnosis records, with first-line treatment starts spanning 2013–2025.

The RECAPC registry protocol was reviewed and approved by the research ethics committee at each participating institution before patient data were incorporated into the registry. The present analysis was prespecified in the registry dissemination plan. Participating sites included 11 institutions across 5 Colombian cities. In accordance with Colombian Resolution 8430 of 1993 (20), the project was classified as research without risk because it used de-identified registry data and involved no intervention or direct patient contact; informed consent was therefore waived by the ethics committees of the participating institutions.

### Participants and cohort definition

The analytic population was restricted to patients with metastatic disease at diagnosis (stage IVA/IVB or metastatic coding at diagnosis). Follow-up and time-to-event analyses used endpoint-specific evaluable subsets defined *a priori* from available dates in the frozen extract.

Study size was determined by all eligible metastatic-at-diagnosis records available at the registry cutoff; no formal sample size calculation was performed for this registry-based analysis.

Metastatic burden was operationalized as the number of involved organs or organ systems documented in the registry. For multivariable modeling, metastatic burden was categorized as >3 versus ≤3 involved organs to preserve interpretability and avoid sparse categories. Organ-specific metastatic site fields were used for consistency checks when available. This measure should be interpreted as a registry-based count of involved organs rather than as radiologic tumor volume, number of individual lesions, or a site-specific prognostic score. The registry captured staging and metastatic involvement, but imaging modalities used for staging were not recorded in a standardized field.

### Molecular variables and driver grouping

Molecular results included epidermal growth factor receptor (EGFR), anaplastic lymphoma kinase (ALK), and ROS1, along with an “other mutation” field supported by free-text specification. Because a patient could have more than one recorded alteration, we separated non-exclusive molecular positivity counts used for quality control from a mutually exclusive driver grouping used for stratified analyses. The mutually exclusive driver cascade assigned each patient to one of four groups: EGFR or ALK altered, other actionable altered (including ROS1 alterations and other conservatively defined actionable findings), no driver detected, or not tested/unknown. “Other actionable” alterations were identified conservatively from free-text entries to avoid inference beyond what was documented. Other named molecular alterations that did not meet the conservative actionable definition were retained as descriptive free-text findings and were not automatically incorporated into the actionable-driver cascade.

Recorded molecular testing type was captured as no test performed, polymerase chain reaction (PCR), immunohistochemistry (IHC), fluorescence *in situ* hybridization (FISH), next-generation sequencing (NGS), or not completed. This variable was used descriptively to summarize the assay type recorded in the registry. The field did not consistently capture assay breadth, such as hotspot testing versus broad comprehensive genomic profiling, nor sample source, such as liquid biopsy versus FFPE tissue. Quality-control review identified records coded as no test performed despite documented molecular results; biomarker results were retained as recorded, and this discrepancy was interpreted as inconsistency in the testing-type field rather than as absence of molecular information.

PD-L1 expression was summarized as <1%, 1–49%, ≥50%, not done, or no data using the registry category field available in the updated extract.

### First-line treatment classification

First-line systemic therapy was classified from line 1 regimen documentation using a deterministic hierarchy to assign one of five manuscript categories: targeted therapy, chemotherapy alone, chemo-immunotherapy, immunotherapy alone, and not reported. “Not reported” reflected missing regimen documentation in the registry extract and was kept distinct from “no systemic therapy” to avoid misclassifying non-reporting as a clinical state. Among patients classified as receiving first-line targeted therapy, regimen text was reviewed descriptively to classify targeted agents by generation when the drug name was identifiable. This supplemental classification did not alter the primary first-line treatment categories.

### Outcomes

Overall survival was defined from a prespecified time origin (typically first-line start) to death; patients without a recorded death were censored at last documented contact. The overall survival analysis set comprised 490 patients with valid time origin and end-of-follow-up dates, with 229 deaths. Because progression dates were incomplete, additional treatment-trajectory endpoints were operationalized using treatment-line dates, including real-world time to next treatment (based on second-line start when available) and real-world time to treatment discontinuation for first-line therapy (based on documented end-of-line timing).

Safety and tolerability variables were recorded as binary indicators (0/1) of adverse events and organ-system toxicities by treatment line. Because the extract did not include Common Terminology Criteria for Adverse Events (CTCAE) grade, serious adverse event classification, or formal treatment attribution, safety analyses were descriptive and intended to characterize recorded events rather than treatment-related risk.

### Statistical analysis

Baseline characteristics were summarized using standard descriptive statistics. Categorical variables are reported as counts and percentages, and continuous variables as means with standard deviations or medians with interquartile ranges, as appropriate. Missingness was reported at the variable level.

Supplemental descriptive analyses summarized recorded molecular testing type, named other-mutation entries, the conservative subset of other actionable alterations, targeted-therapy agent generation among patients receiving first-line targeted therapy, metastatic burden distribution, and availability of staging-imaging modality information. These analyses were descriptive and did not modify the prespecified driver cascade, first-line treatment categories, survival endpoints, or multivariable models.

Time-to-event analyses focused on overall survival and operational treatment-trajectory endpoints derived from the registry. Overall survival was estimated with the Kaplan–Meier method, and follow-up time with the reverse Kaplan–Meier approach. Analyses were performed in prespecified evaluable subsets defined by the availability of the required time-origin and follow-up dates. No imputation was performed.

Associations between clinical variables and overall survival were examined with multivariable Cox proportional hazards models including driver group and prespecified covariates available in the locked analytic dataset. Covariates included age, sex, Eastern Cooperative Oncology Group performance status, metastasis burden, insurance group, histology group, and PD-L1 category. PD-L1 was modeled as a five-level categorical variable (<1%, 1–49%, ≥50%, not done, and no data), with <1% as the reference category. Effect estimates are reported as hazard ratios with 95% confidence intervals. Age was modeled continuously and is presented as the hazard ratio per 10-year increase to improve clinical interpretability. No imputation was performed; therefore, multivariable models were fitted on complete cases only. Proportional hazards assumptions were assessed using standard diagnostic procedures.

Registry placeholders indicating non-reporting were treated as missing during data harmonization and were not reinterpreted as clinical categories. When registry fields explicitly distinguished categories such as “no data” or “not done,” those levels were retained as recorded. Prespecified variable definitions, use of a locked analytic dataset, and explicit separation of “not reported” from clinical treatment categories were used to reduce misclassification.

Safety and tolerability variables were analyzed descriptively because the extract stored these fields as binary indicators by treatment line and did not include CTCAE grade, serious adverse event classification, or treatment attribution.

All analyses were conducted in R (version 4.5.2) (21) using a scripted workflow that generated a single integrated report containing all tables and figures. Manuscript outputs were produced from a locked analytic dataset accompanied by a SHA-256 manifest for file integrity verification. Prespecified quality-control procedures were applied before report generation to confirm the internal consistency of key denominators, derived variables, and time-to-event inputs.

## Results

**Figure 1:**
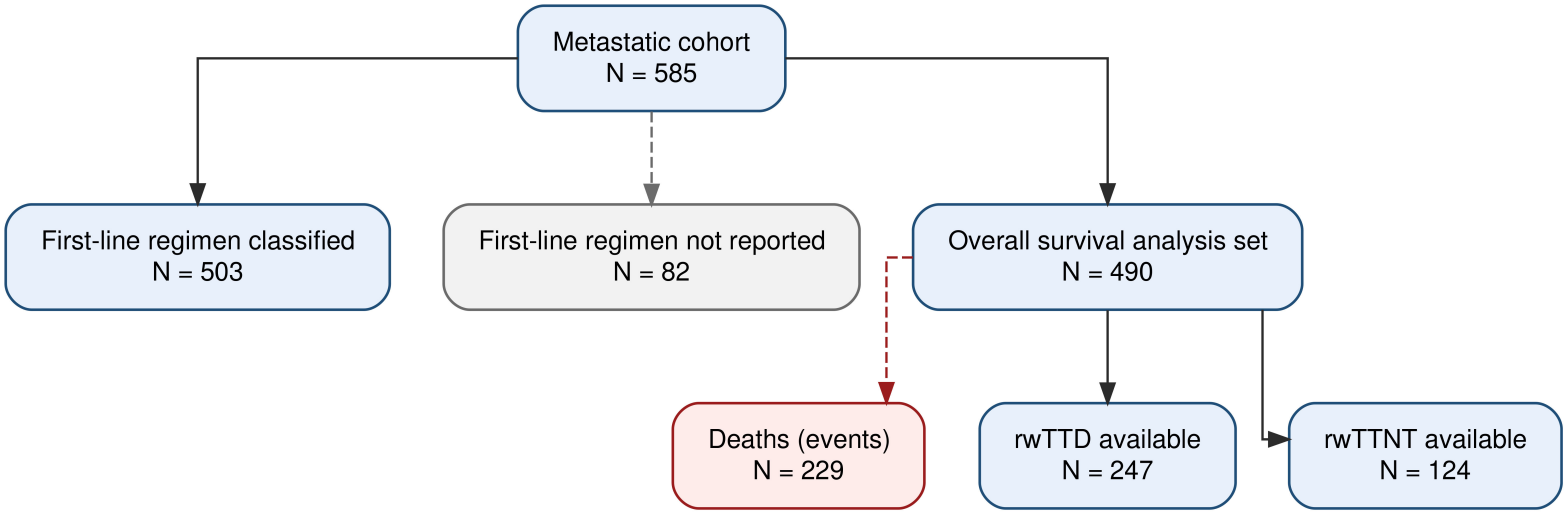
Study flow and analysis denominators in the RECAPC metastatic cohort. Flow diagram showing the metastatic cohort (N = 585), the first-line classifiable denominator for five regimen categories (N = 503; not reported N = 82), the overall survival analysis set (N = 490; deaths N = 229), and availability of operational treatment-trajectory endpoints (real-world time to next treatment N = 124; real-world time to treatment discontinuation N = 247). “Not reported” indicates missing regimen documentation in the source extract.

The metastatic-at-diagnosis cohort included 585 patients with metastatic non–small cell lung cancer. Of these, 509 had a valid time origin date for time-to-event analyses, and 490 met prespecified criteria for overall survival evaluation, contributing 229 deaths. Operational treatment-trajectory endpoints were available for 124 patients for real-world time to next treatment and 247 for real-world time to treatment discontinuation. Participant flow and analysis denominators are summarized in **Figure 1**.

### Baseline characteristics

Baseline characteristics are presented in **Table 1** with variable-level missingness. Mean (SD) age was 72.0 (16.0) years; 304 patients (52.0%) were female. Most patients had non-squamous histology (450, 76.9%) and contributory insurance (412, 70.4%). Smoking history was recorded as never in 278 (47.5%), former in 226 (38.6%), current in 56 (9.6%), and passive exposure in 25 (4.3%). The median metastatic burden was 2 involved organs (IQR, 1–3); 523 patients (89.4%) had involvement of ≤3 organs, and 62 (10.6%) had involvement of >3 organs.

**Table 1:** Baseline characteristics and key clinical variables (N = 585).

Characteristic	Overall
*Age, years, mean (SD)*	72.0 (16.0)*
*Female sex, n (%)*	304 (52.0)
Insurance group, n (%)	
Contributory	412 (70.4)
Subsidized	160 (27.4)
Other	13 (2.2)
*ECOG performance status, median (IQR)*	1.0 (0.0)*
Histology group, n (%)	
Non-squamous	450 (76.9)
Squamous	91 (15.6)
Not otherwise specified/other	44 (7.5)
Smoking status, n (%)	
Never	278 (47.5)
Former	226 (38.6)
Current	56 (9.6)
Passive	25 (4.3)
*Metastasis burden (number of involved organs), median (IQR)*	2 (1–3)
Metastasis burden ≤3 involved organs, n (%)	523 (89.4)
Metastasis burden >3 involved organs, n (%)	62 (10.6)
PD-L1 category, n (%)	
<1%	149 (25.5)
1–49%	119 (20.3)
≥50%	74 (12.6)
Not done	156 (26.7)
No data	87 (14.9)
Driver group (mutually exclusive cascade), n (%)	
EGFR or ALK altered	192 (32.8)
Other actionable altered	27 (4.6)
No driver detected	88 (15.0)
Not tested/unknown	278 (47.5)
First-line regimen category, n (%)	
Chemotherapy alone	201 (34.4)
Chemo-immunotherapy	123 (21.0)
Immunotherapy alone	23 (3.9)
Targeted therapy	156 (26.7)
Not reported	82 (14.0)

Values are n (%) unless otherwise specified. *Missingness: age missing in 1 patient (0.2%); ECOG missing in 1 patient (0.2%). “Not reported” indicates missing regimen documentation and is not interpreted as absence of treatment. ECOG indicates Eastern Cooperative Oncology Group; EGFR, epidermal growth factor receptor; ALK, anaplastic lymphoma kinase; PD-L1, programmed death-ligand 1.

Programmed death-ligand 1 status was categorized as <1% in 149 patients (25.5%), 1–49% in 119 (20.3%), and ≥50% in 74 (12.6%); testing was not done in 156 (26.7%), and data were missing in 87 (14.9%). Non-exclusive documented biomarker positives included EGFR in 131 patients (22.4%) and ALK in 63 (10.8%). The mutually exclusive driver cascade classified 192 (32.8%) as EGFR or ALK altered, 27 (4.6%) as Other actionable altered, 88 (15.0%) as no driver detected, and 278 (47.5%) as not tested/unknown. First-line therapy was categorized as chemotherapy alone in 201 (34.4%), chemo-immunotherapy in 123 (21.0%), immunotherapy alone in 23 (3.9%), targeted therapy in 156 (26.7%), and not reported in 82 (14.0%).

### Molecular alterations and first-line treatment patterns

Molecular alteration counts used for quality control (non-exclusive) included EGFR positive in 131 patients, ALK positive in 63, and ROS1 positive in 15. The other-mutation free-text field contained named entries in 48 patients. Using the conservative prespecified rule applied in the locked pipeline, 17 patients were classified as having another actionable alteration; the patient-level summary and clinically interpretable free-text groups are provided in **Supplementary Table 1**. PD-L1 expression was <1% in 149 patients (25.5%), 1–49% in 119 (20.3%), and ≥50% in 74 (12.6%); among 342 patients with an interpretable PD-L1 result, 193 (56.4%) had PD-L1 expression ≥1% and 74 (21.6%) had PD-L1 expression ≥50%.

**Figure 2:**
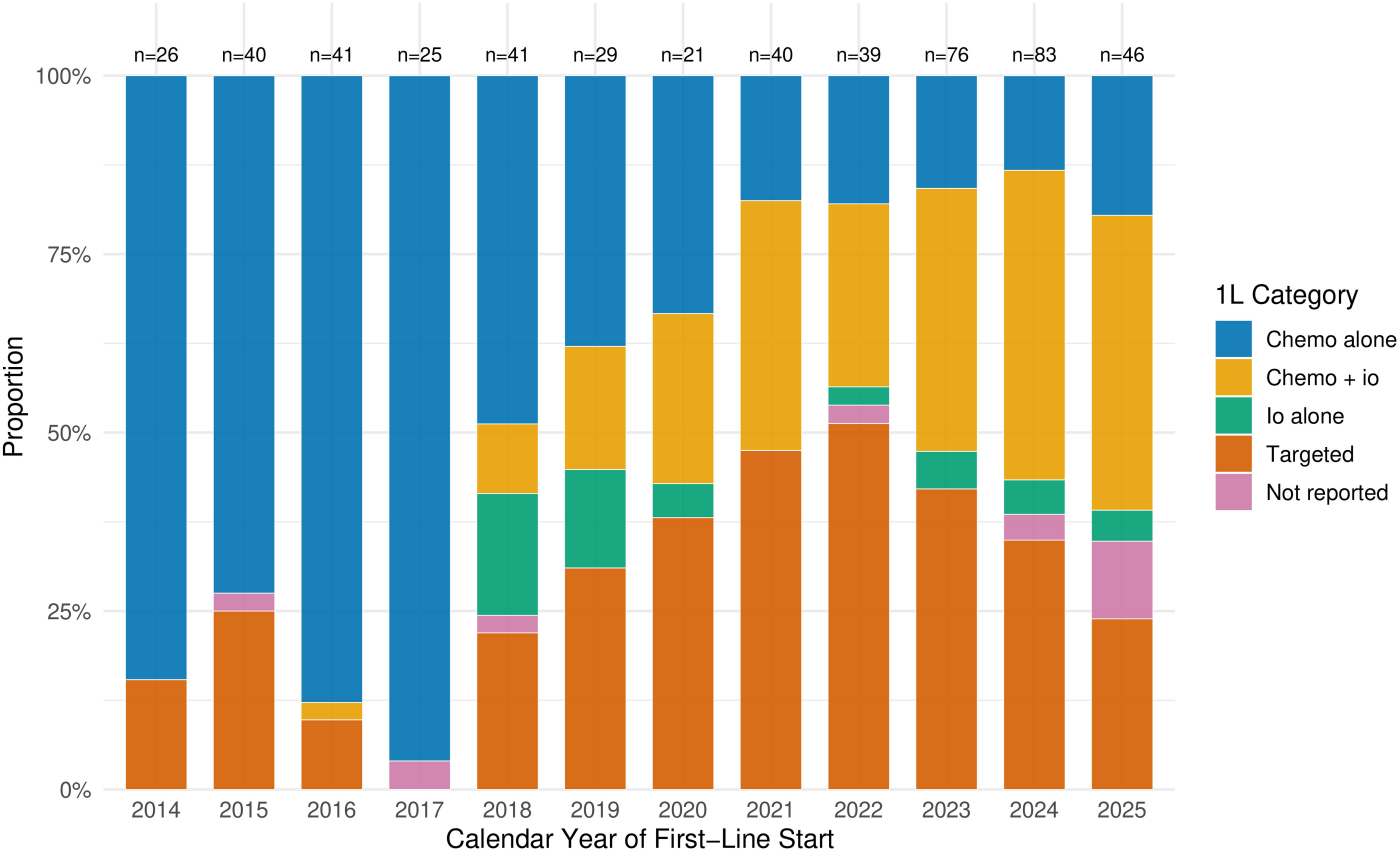
Temporal adoption of first-line regimen categories. One-hundred percent stacked bar chart showing the distribution of five first-line regimen categories by calendar year of first-line start. Years with fewer than five patients are excluded. Categories are chemotherapy alone, chemo-immunotherapy, immunotherapy alone, targeted therapy, and not reported.

Recorded molecular testing type was NGS in 226 patients (38.6%), PCR in 121 (20.7%), IHC in 121 (20.7%), FISH in 5 (0.9%), and no test performed in 112 (19.1%); no records had this field left blank. Quality-control review identified 49 patients coded as having no test performed despite documented molecular results. These biomarker results were retained as recorded, and the discrepancy was interpreted as inconsistency in the testing-type field. The registry did not consistently distinguish hotspot testing from broad comprehensive genomic profiling or liquid biopsy from FFPE tissue. **Figure 2** shows the temporal adoption of first-line regimen categories between 2014 and 2025, with a progressive decline in chemotherapy alone after 2018 and a corresponding increase in immunotherapy-based and targeted-therapy regimens.

**Figure 3:**
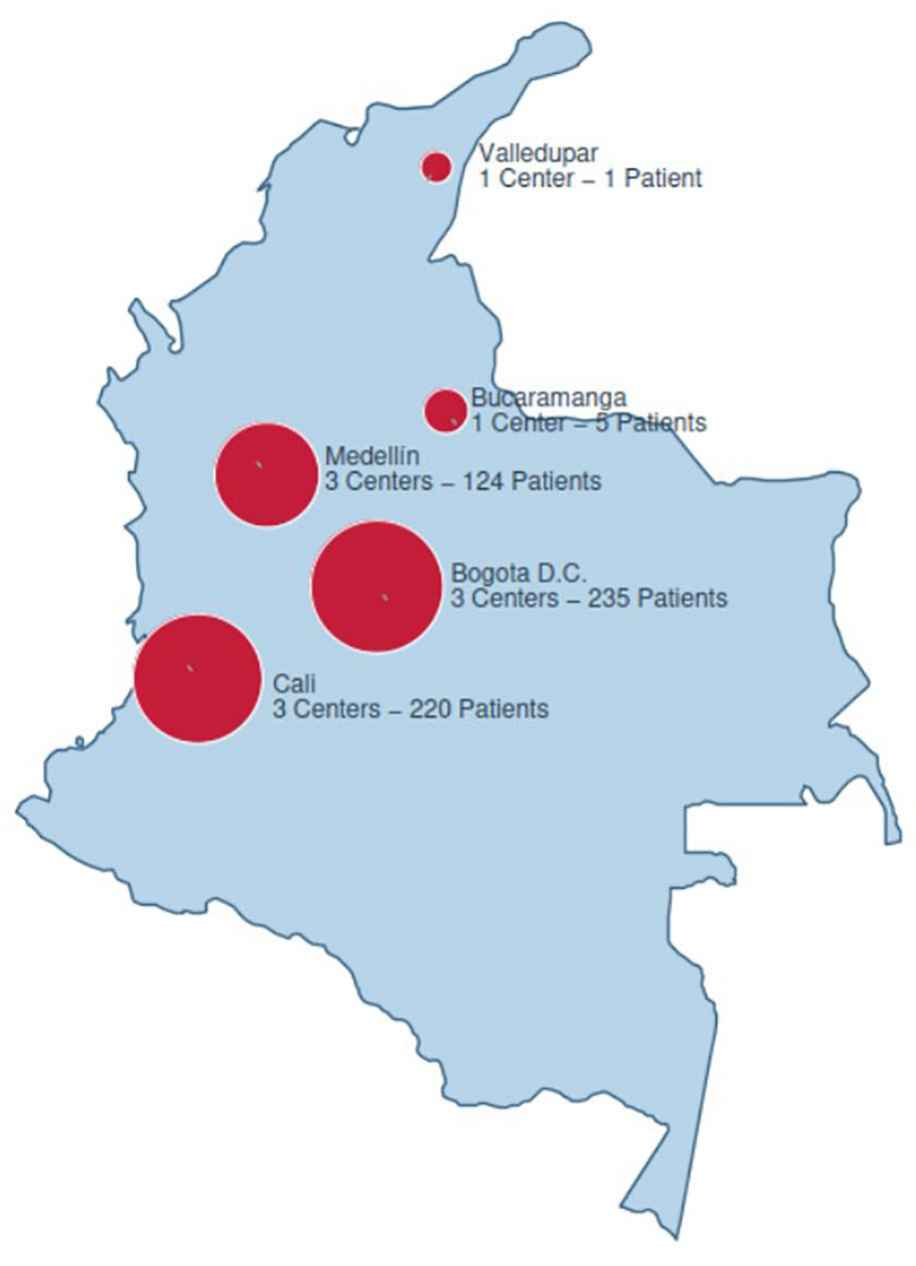
Geographic distribution of contributing centers in Colombia. Map of Colombia displaying participating registry centers by city. Point size reflects the number of patients contributed by each city.

The mutually exclusive cascade distribution is shown in **Table 2**, and the cross-tabulation of driver group by first-line regimen category is shown in **Table 3**. The geographic distribution of contributing centers is shown in **Figure 3**.

**Table 2:** Molecular status summary: driver cascade and non-exclusive documented positives (N = 585).

Metric	n (%)
Driver group (mutually exclusive cascade)	
EGFR or ALK altered	192 (32.8)
Other actionable altered	27 (4.6)
No driver detected	88 (15.0)
Not tested/unknown	278 (47.5)
QC (non-exclusive documented positives)	
EGFR positive	131 (22.4)
ALK positive	63 (10.8)
ROS1 positive	15 (2.6)
Other mutation with named free-text entry	48 (8.2)
Other actionable by conservative free-text rule	17 (2.9)

QC counts are non-exclusive documented positives derived from the full cohort (N = 585). Other mutation with named free-text entry indicates any specified non-EGFR/ALK/ROS1 alteration recorded in the free-text field. Other actionable by conservative free-text rule indicates the subset retained as actionable according to the prespecified conservative parsing rule. PD-L1 is reported separately in the manuscript as <1%, 1–49%, ≥50%, not done, and no data.

**Table 3:** First-line regimen category within driver groups (classifiable denominator N = 503).

Driver group	Targeted	Chemo-immunotherapy	Immunotherapy alone	Chemotherapy alone	Total
EGFR or ALK altered	140	2	0	37	179
Other actionable altered	6	13	1	6	26
No driver detected	0	55	10	15	80
Not tested/unknown	10	53	12	143	218
Total	**156**	**123**	**23**	**201**	**503**

**Table 3** excludes patients with first-line regimen categorized as “not reported” (N = 82), which are described in **Table 1**. Bold values indicate column totals for the classifiable first-line regimen denominator.

The registry captured stage and metastatic involvement, but imaging modalities used for staging were not recorded in a standardized field. Therefore, the cohort could not be summarized by CT, PET-CT, brain MRI, bone scan, or other staging-imaging modality.

Among 156 patients classified as receiving first-line targeted therapy, regimen text allowed classification by agent generation. EGFR third-generation therapy accounted for 76 patients (48.7%), later-generation ALK therapy for 35 (22.4%), EGFR first- or second-generation therapy for 28 (17.9%), first-generation ALK therapy for 15 (9.6%), and other named targeted agents for 2 (1.3%). This supplemental description was intended to characterize practice patterns and did not modify the primary first-line treatment categories. Targeted-therapy generation groups are provided in **Supplementary Table 2**.

### Treatment trajectories by first-line category

**Figure 4:**
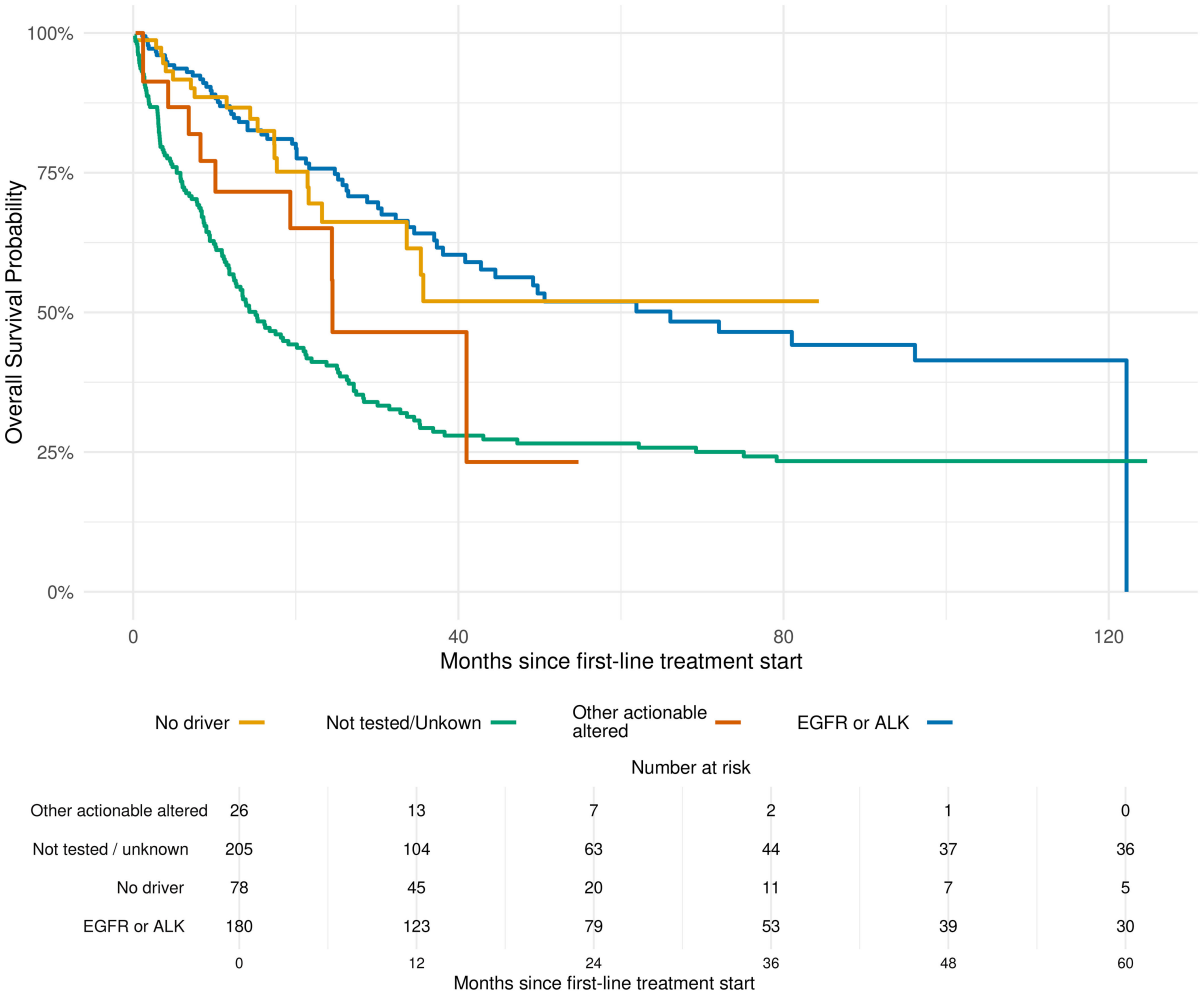
Sankey diagram linking driver group, first-line category, and outcome state. Three-stage Sankey plot showing patient counts across driver group (mutually exclusive cascade), first-line regimen category (five-category manuscript version), and outcome state (still on first-line therapy, switched to second-line therapy, death, censored).

Outcome states derived from treatment-line documentation are summarized in **Table 4**. Across first-line categories, deaths accounted for 134 of 201 (66.7%) in the chemotherapy-alone group, 43 of 123 (35.0%) in the chemo-immunotherapy group, 11 of 23 (47.8%) in the immunotherapy-alone group, 59 of 82 (72.0%) among patients with not-reported first-line regimens, and 49 of 156 (31.4%) in the targeted-therapy group. The three-stage flow from driver group to first-line category to outcome state is shown in **Figure 4**.

**Table 4:** Outcomes by first-line regimen category.

First-line regimen category	N	Censored, n (%)	Death, n (%)	Still on first-line, n (%)	Switched to second-line, n (%)
Chemotherapy alone	201	2 (1.0%)	134 (66.7%)	38 (18.9%)	27 (13.4%)
Chemo-immunotherapy	123	1 (0.8%)	43 (35.0%)	66 (53.7%)	13 (10.6%)
Immunotherapy alone	23	0 (0.0%)	11 (47.8%)	12 (52.2%)	0 (0.0%)
Not reported	82	14 (17.1%)	59 (72.0%)	7 (8.5%)	2 (2.4%)
Targeted therapy	156	0 (0.0%)	49 (31.4%)	92 (59.0%)	15 (9.6%)

Percentages are row percentages within each first-line category. “Censored” indicates alive at last recorded contact without death in the survival dataset.

### Safety and tolerability

Safety variables were available as binary indicators by treatment line. One patient had a recorded cutaneous adverse event during first-line targeted therapy, corresponding to 1 of 509 patients with evaluable first-line safety data (0.2%) and 1 of 156 patients in the targeted-therapy group (0.6%). One additional positive second-line binary safety flag was recorded (1/124, 0.8%), and no third-line events were documented. Hematologic toxicity was not available in the extract, and no CTCAE grade, serious adverse event classification, or formal treatment attribution was recorded. Given the extremely low event frequency, these fields were interpreted as incomplete safety capture rather than evidence of minimal treatment toxicity.

### Follow-up and survival outcomes

**Figure 5:**
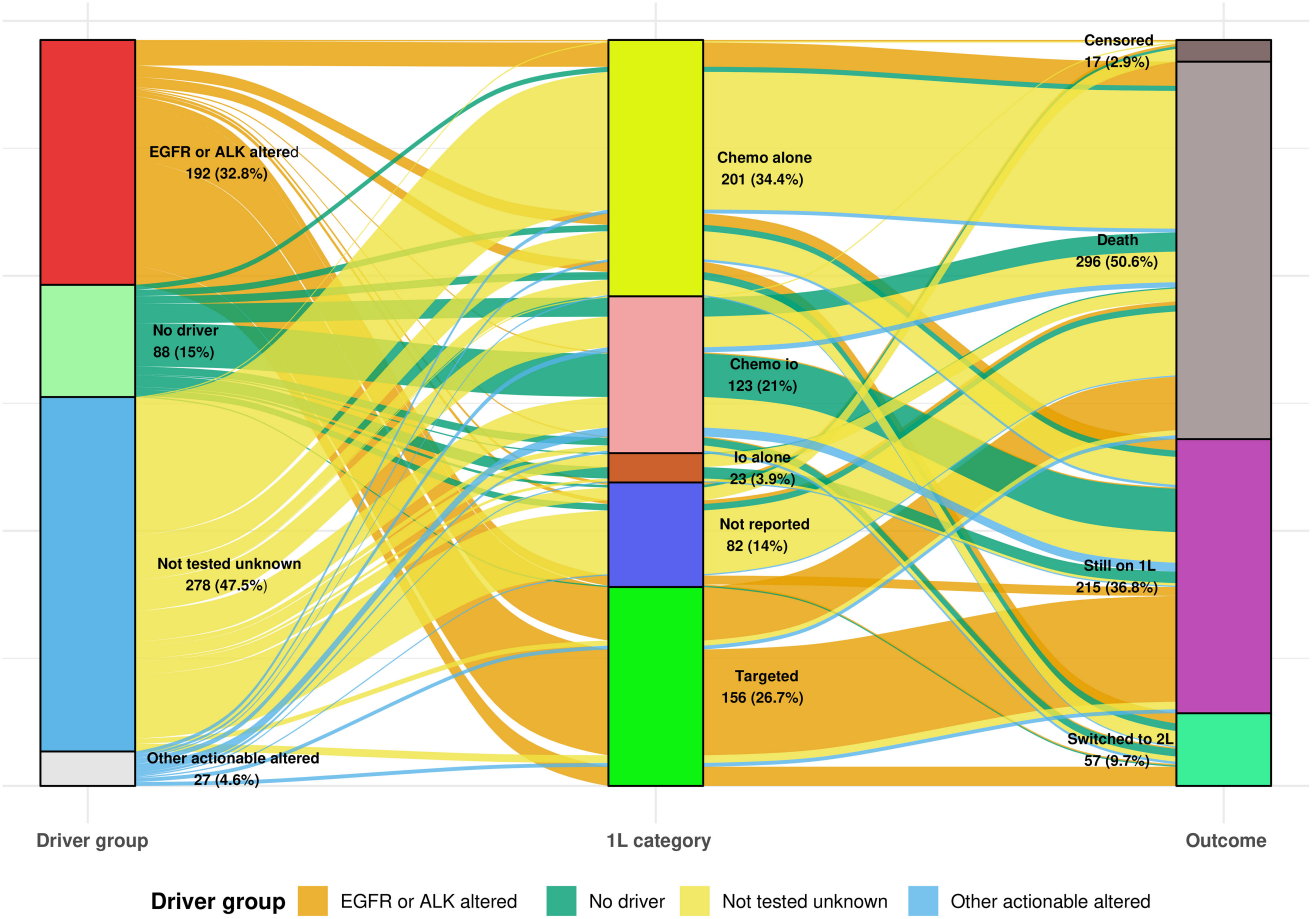
Kaplan–Meier overall survival by mutually exclusive driver group. Kaplan–Meier overall survival curves stratified by the mutually exclusive driver cascade (EGFR or ALK altered; Other actionable altered; no driver detected; not tested/unknown) in the overall survival analysis set (n = 490; deaths n = 229).

Median follow-up estimated from the extract using reverse Kaplan–Meier methods was 34.99 months in the overall survival analysis set (n = 490). Overall survival Kaplan–Meier curves by driver group are shown in **Figure 5** (log-rank P < 0.001). Survival probability estimates at 1, 3, and 5 years by driver group are presented in **Table 5**.

**Table 5:** Kaplan–Meier overall survival point estimates by driver group.

Driver group	12-month survival (95% CI)	n at risk	36-month survival (95% CI)	n at risk	60-month survival (95% CI)	n at risk
EGFR or ALK altered	86.2% (81.0–91.8)	123	64.1% (55.9–73.5)	53	52.0% (42.8–63.1)	30
Other actionable altered	71.6% (54.6–93.9)	13	46.5% (26.2–82.4)	2	23.2% (5.2–100.0)	0
No driver detected	86.7% (78.8–95.3)	45	52.0% (36.8–73.6)	11	52.0% (36.8–73.6)	5
Not tested/unknown	56.6% (50.0–64.0)	104	29.2% (23.1–36.9)	44	26.4% (20.5–34.1)	36

Estimates are Kaplan–Meier survival probabilities. Precision at later time points is limited in strata with small numbers at risk.

### Multivariable overall survival model

Adjusted hazard ratios for overall survival are shown in **Table 6**. In the complete-case multivariable Cox model (n = 489; deaths = 228), the not tested/unknown driver group remained associated with higher mortality relative to EGFR or ALK altered (hazard ratio 2.26; 95% CI, 1.62–3.16; P < 0.001). Age, reported per 10-year increase, was also associated with higher mortality (hazard ratio 1.28; 95% CI, 1.10–1.48; P < 0.001). Compared with Eastern Cooperative Oncology Group performance status 0, performance status 2 remained associated with higher mortality (hazard ratio 1.76; 95% CI, 1.10–2.81; P = 0.02). Using PD-L1 <1% as the reference category, hazard ratios were 0.93 (95% CI, 0.61–1.41; P = 0.72) for 1–49%, 0.98 (95% CI, 0.62–1.56; P = 0.94) for ≥50%, 0.80 (95% CI, 0.54–1.17; P = 0.25) for not done, and 1.33 (95% CI, 0.83–2.13; P = 0.23) for no data. The operational progression-free survival Cox model was not estimable in this dataset because of insufficient variation in the endpoint definition.

**Table 6:** Multivariable Cox proportional hazards model for overall survival (n = 489; deaths = 228).

Covariate	Hazard ratio	95% CI	P value
Driver group (ref: EGFR or ALK altered)			
Other actionable altered	1.65	0.83–3.28	0.15
No driver detected	1.03	0.60–1.76	0.90
Not tested/unknown	2.26	1.62–3.16	<0.001
Age (10-year increase)	1.28	1.10–1.48	<0.001
Male sex (ref: female)	1.06	0.80–1.40	0.68
ECOG performance status (ref: 0)			
1	1.01	0.69–1.47	0.95
2	1.76	1.10–2.81	0.02
3	1.41	0.66–3.00	0.37
4	1.31	0.39–4.42	0.67
Metastatic burden >3 organs (ref: ≤3)	1.46	0.95–2.26	0.09
Insurance (ref: contributory)			
Other	1.06	0.47–2.39	0.90
Subsidized	1.02	0.74–1.41	0.89
Histology (ref: non-squamous)			
Not otherwise specified/other	1.04	0.56–1.91	0.91
Squamous	1.11	0.75–1.63	0.61
PD-L1 category (ref: <1%)			
1–49%	0.93	0.61–1.41	0.72
≥50%	0.98	0.62–1.56	0.94
Not done	0.80	0.54–1.17	0.25
No data	1.33	0.83–2.13	0.23

CI indicates confidence interval; ECOG, Eastern Cooperative Oncology Group; PD-L1, programmed death-ligand 1. Age was modeled continuously and is presented per 10-year increase for interpretability.

## Discussion

This multicenter Colombian cohort provides a real-world description of molecular documentation, first-line treatment patterns, and survival in metastatic-at-diagnosis non–small cell lung cancer. Two features shaped the interpretation of most analyses: nearly half of patients were categorized as not tested/unknown by the prespecified driver cascade, and 14% had first-line regimen documentation classified as not reported. First-line therapy spanned chemotherapy alone, chemo-immunotherapy, immunotherapy alone, and targeted therapy, with clear calendar-time shifts in regimen mix. Overall survival differed across driver groups, and in adjusted analyses the not tested/unknown category was associated with higher mortality risk than the EGFR/ALK-altered group. These associations describe patterns in routine care and should not be read as treatment effects.

The not tested/unknown category should be interpreted as a composite registry state rather than as a single biologic subgroup. It may include patients who were not tested, patients whose results were not transferred into structured registry fields, and patients whose diagnostic pathway was interrupted or incompletely documented. From a prognostic-factor risk-of-bias perspective, this distinction matters because the category combines molecular uncertainty, measurement limitations, and residual confounding by access to care. Prior population-based studies have shown that socioeconomic factors are associated with NSCLC treatment patterns, stage distribution, and survival outcomes (22, 23). In the Colombian setting, payer authorization processes, fragmented provider networks, geographic concentration of oncology services, and referral delays may influence whether molecular testing is performed, whether results are available when treatment is selected, and whether biomarker-directed therapy can be delivered. The higher mortality observed in the not tested/unknown group should therefore be read as an observational signal of a vulnerable care pathway, not as the isolated effect of absent molecular testing.

The finding that 14.0% of patients were classified as “not reported” for first-line regimen documentation should be interpreted within the structural realities of cancer care in Colombia. Qualitative work in public healthcare networks has identified insurer authorization processes and fragmented provider contracting as major sources of delay in the diagnostic and treatment pathway (15). Geographic concentration of oncology services and interregional travel burdens add to these discontinuities (16, 17). In that context, a missing regimen entry in a multicenter registry can reflect incomplete transfer across provider networks, delays in treatment delivery, or both, rather than a purely clerical omission (18, 19).

### Biomarker documentation and its clinical implications in routine care

Incomplete biomarker characterization is common in real-world datasets and reflects a mix of access constraints, tissue limitations, turnaround time, and incomplete capture of results in structured fields (8, 9). In this cohort, recorded assay type provided additional context: NGS was the most frequently recorded test type, followed by PCR and IHC, with FISH recorded less often. That distribution should be interpreted cautiously. The registry field captured broad assay type, but not assay breadth, sample source, or whether NGS represented broad comprehensive genomic profiling versus a smaller panel. Quality-control review also identified records coded as no test performed despite documented molecular results, consistent with incomplete or inconsistent real-world documentation. In that context, separating “no driver detected” from “not tested/unknown” remains methodologically important because absence of documentation cannot be interpreted as a negative result.

PD-L1 in this cohort should be interpreted as both a biologic marker and a marker of care processes. A substantial proportion of patients with an interpretable result had PD-L1 expression ≥1%, including a smaller subgroup with PD-L1 ≥50%, which is clinically relevant for first-line treatment selection. In the multivariable model, PD-L1 categories were not independently associated with overall survival relative to <1%. In routine care, that finding is plausible because PD-L1 does not act in isolation: treatment assignment is influenced by driver status, performance status, and access to biomarker-guided therapy, while incomplete PD-L1 ascertainment can still limit timely treatment selection.

### Driver distribution in a regional context

Driver prevalence varies across populations. A meta-analysis in Hispanic/Latino cohorts reported higher EGFR alteration frequency than many non-Latin series and substantial heterogeneity across countries and ancestry backgrounds (10). Regional real-world studies that include Colombia have also documented uneven access to EGFR testing and variable uptake of matched therapy (11, 24). In this analysis, driver status was summarized both as documented positives for quality control and as a mutually exclusive cascade for stratified survival analyses. That structure is useful in registry settings where multiple positives can appear through sequential testing or documentation differences and helps avoid overstating mutually exclusive driver frequencies.

Rare actionable alterations also matter in practice because they are difficult to characterize without pooled datasets. Latin American collaborative work has shown how multicenter real-world data can describe uncommon subtypes such as ERBB2-mutant disease and quantify the surrounding gaps in testing and treatment access (13). Center-based experience from the region similarly suggests that broader genomic profiling can support matched therapy strategies while underscoring that access to both testing and targeted drugs is uneven (12).

### First-line treatment patterns and contemporary standards

Guideline-based first-line selection depends on driver status and PD-L1 expression, with targeted therapy preferred for actionable alterations and immune checkpoint inhibitor–based regimens prioritized for driver-negative disease according to PD-L1 strata (1). Trial benchmarks frame expectations for outcomes when treatment is matched to biology, including long-term pembrolizumab benefit in PD-L1–high disease and improved survival with first-line osimertinib in EGFR-mutant disease (2, 3). In routine care, adoption is paced by drug availability, coverage, and local practice pathways. Observational datasets show that testing and treatment uptake evolve over time, with plateauing of some testing metrics even as systemic options expand (7, 25). The temporal changes observed here should be interpreted as practice-pattern description rather than comparative effectiveness evidence.

The distribution of first-line targeted agents also reflects evolving practice. Among patients receiving first-line targeted therapy, later-generation EGFR and ALK inhibitors accounted for most classified regimens, while first-generation ALK and first- or second-generation EGFR agents remained represented. These data should be interpreted as descriptive practice patterns rather than comparative effectiveness evidence. Treatment generation is shaped by calendar year, biomarker subtype, reimbursement, availability, and physician selection, and the registry was not designed to compare outcomes across individual targeted agents.

Metastatic burden also requires a cautious reading. The registry operationalized this variable as the number of involved organs or organ systems, with >3 organs used as the model category. Most patients had involvement of ≤3 organs, and the >3-organ group represented 10.6% of the cohort. This measure captures extent of documented organ involvement, but it does not capture lesion count, tumor volume, symptomatic burden, or the prognostic weight of specific metastatic sites such as brain, liver, or bone. Its borderline association with overall survival should therefore be interpreted as a coarse marker of disease extent rather than as a complete measure of metastatic risk.

### Survival patterns: cautious interpretation

Survival differences across driver groups are directionally consistent with the clinical expectation that timely biomarker identification and access to matched therapy coincide with better outcomes. At the same time, survival comparisons in real-world cohorts are strongly influenced by baseline status, access, and documentation completeness. Performance status is a dominant prognostic factor in immunotherapy-treated populations, and patients with ECOG performance status ≥2 have substantially poorer outcomes in real-world cohorts and meta-analyses (26–28). The higher hazard observed in the not tested/unknown group likely reflects confounding by baseline risk and access rather than an effect of testing itself. Because multivariable estimates were derived from complete-case analysis, they should be interpreted within the subset of patients with complete covariate information.

Comparisons between real-world outcomes and trial benchmarks also require restraint. In PD-L1–high disease, real-world 5-year pembrolizumab outcomes are lower than trial results, consistent with broader eligibility and higher baseline risk in practice (2, 29). Those benchmarks help contextualize expectations but do not establish causal explanations for differences across cohorts.

### Treatment-trajectory endpoints when progression is incompletely captured

Progression dates are often missing in registries, and treatment-line transitions or discontinuation are frequently used as operational proxies for disease control (30). Time to treatment discontinuation is widely used in lung cancer real-world research and shows correlation with progression-free survival across published experience, while remaining sensitive to non-progression drivers of treatment change such as toxicity, preference, and access disruptions (31). In this cohort, these operational endpoints were available in subsets of patients, supporting treatment-trajectory description but limiting precision for subgroup inference. Post–immune checkpoint inhibitor care is heterogeneous, and downstream outcomes depend on selection into subsequent therapy and baseline risk (32–34).

Safety interpretation was limited because the registry stored binary indicators by treatment line rather than graded adverse events. Only one cutaneous adverse event was recorded, without CTCAE grade, serious adverse event classification, or formal treatment attribution. This pattern is more consistent with undercapture in routine documentation than with absence of treatment-related toxicity.

### Strengths, limitations, and generalizability

This study provides a multicenter snapshot of real-world care, but its findings should be interpreted within the limits of registry-based observational data. Treatment selection was not randomized, so confounding by indication, baseline risk, and access is expected. Missingness and incomplete capture can bias subgroup estimates; the likely direction is toward worse observed outcomes in “unknown” categories because these strata can concentrate limited access, poorer baseline status, and incomplete follow-up. The not tested/unknown category is especially vulnerable to this problem because it may combine biologic uncertainty, incomplete documentation, delayed testing, and access-related barriers rather than a single uniformly measured prognostic factor. Separately, incomplete regimen documentation can misclassify treated patients as “not reported,” which can dilute comparisons across first-line categories.

Several requested variables were available only with limited granularity. The registry captured recorded assay type, but it did not consistently distinguish hotspot testing from broad comprehensive genomic profiling or liquid biopsy from tissue-based testing. Imaging modalities used for staging were not recorded in a standardized field. Metastatic burden was measured as the number of involved organs, not as radiologic tumor volume, lesion count, or site-specific metastatic risk. Safety interpretation was limited by binary event indicators without CTCAE grade, serious adverse event classification, or formal treatment attribution.

Generalizability also requires restraint. Because RECAPC includes patients treated in participating oncology centers, the cohort may underrepresent patients managed outside specialized cancer pathways or those with greater barriers to referral, diagnostic completion, molecular testing, or systemic treatment. The observed survival disadvantage in the not tested/unknown group may therefore be conservative relative to the broader Colombian metastatic NSCLC population. The most defensible external validity is to institutions and care pathways with comparable diagnostic resources, payer structure, and treatment availability (5, 11).

## Conclusions

In the RECAPC registry cohort of patients with metastatic-at-diagnosis non–small cell lung cancer (N = 585), incomplete molecular characterization and missing first-line regimen documentation were common. Among patients evaluable for overall survival (n = 490), there were 229 deaths and a median follow-up of 34.99 months. First-line treatment patterns spanned chemotherapy alone, chemo-immunotherapy, immunotherapy alone, and targeted therapy, with temporal shifts across calendar years. In this setting, missing regimen documentation should be interpreted cautiously because it may reflect both incomplete capture and discontinuities across fragmented care pathways.

Overall survival differed across driver groups in unadjusted analyses, and in the multivariable Cox model the not tested/unknown group had a higher hazard of death compared with the EGFR/ALK-altered group (HR 2.26; 95% CI, 1.62–3.16). Older age and ECOG performance status 2 were also associated with higher mortality risk. These results describe real-world care and outcomes as documented in the registry and should be interpreted as observational associations rather than causal effects. The not tested/unknown category may also identify patients in more vulnerable diagnostic and care pathways, where incomplete molecular characterization, fragmented documentation, and access barriers overlap.

## External review

An external scientific review of a preliminary draft was conducted, and feedback was incorporated into the final manuscript.

## Data Availability

The data analyzed in this study is subject to the following licenses/restrictions: The datasets analyzed in this study belong to the RECAPC registry and are administered by ACHO. They are not publicly available. Access is considered only upon submission of a justified research request and is subject to ACHO approval and the bioethical and institutional restrictions applicable to each study. Requests to access these datasets should be directed to gerente@acho.com.co.
