# Supplementary material for: Molecular testing, first-line treatment patterns, and survival in metastatic Colombian non–small cell lung cancer: the RECAPC multicenter registry
Source: Front Oncol. 2026 Jul 17;16:1863940. doi: 10.3389/fonc.2026.1863940 (PMC13423713; doi:10.3389/fonc.2026.1863940)
Supplement: Supplementary file 1 [file Table1.docx]

**SUPPLEMENTARY MATERIAL**

**Supplementary Table 1. Other documented mutations and conservative actionable classification.**

| Panel | Category | n | % |
| --- | --- | --- | --- |
| A. Patient-level summary | Patients with any named other-mutation free-text entry | 48 | 8.2% of total cohort |
| A. Patient-level summary | Patients classified as having another actionable alteration by the conservative pipeline rule | 17 | 2.9% of total cohort; 35.4% of patients with named other-mutation entry |
| A. Patient-level summary | Patients with named other-mutation entry not classified as actionable by the conservative pipeline rule | 31 | 5.3% of total cohort; 64.6% of patients with named other-mutation entry |
| B. Clinically interpretable free-text groups | BRAF-related entries | 7 | 14.6% of patients with named other-mutation entry |
| B. Clinically interpretable free-text groups | KRAS-related entries | 7 | 14.6% of patients with named other-mutation entry |
| B. Clinically interpretable free-text groups | EGFR uncommon/amplification-related entries | 7 | 14.6% of patients with named other-mutation entry |
| B. Clinically interpretable free-text groups | MET-related entries | 5 | 10.4% of patients with named other-mutation entry |
| B. Clinically interpretable free-text groups | FGFR/FGF-related entries | 4 | 8.3% of patients with named other-mutation entry |
| B. Clinically interpretable free-text groups | BRCA-related entries | 3 | 6.2% of patients with named other-mutation entry |
| B. Clinically interpretable free-text groups | RET-related entries | 2 | 4.2% of patients with named other-mutation entry |
| B. Clinically interpretable free-text groups | NTRK-related entries | 2 | 4.2% of patients with named other-mutation entry |
| B. Clinically interpretable free-text groups | STK11-related entries | 2 | 4.2% of patients with named other-mutation entry |
| B. Clinically interpretable free-text groups | KEAP1-related entries | 1 | 2.1% of patients with named other-mutation entry |
| B. Clinically interpretable free-text groups | PIK3CA-related entries | 1 | 2.1% of patients with named other-mutation entry |
| B. Clinically interpretable free-text groups | ALK fusion recorded in the other-mutation field | 1 | 2.1% of patients with named other-mutation entry |
| B. Clinically interpretable free-text groups | ERBB2/HER2-related entries | 0 | 0.0% of patients with named other-mutation entry |
| B. Clinically interpretable free-text groups | TMB/MSI/MSS or microsatellite-status entries | 17 | 35.4% of patients with named other-mutation entry |
| B. Clinically interpretable free-text groups | TP53/p53-related entries | 9 | 18.8% of patients with named other-mutation entry |

*Percentages in Panel A use the full metastatic cohort (N = 585) or patients with a named other-mutation free-text entry (N = 48), as indicated. Panel B summarizes clinically interpretable groups derived from cleaned free-text entries. These groups are descriptive and not mutually exclusive; a single free-text entry could include more than one alteration or nonstandard notation. The conservative actionable classification refers only to the prespecified pipeline rule used in the locked analysis. These supplemental counts did not modify the mutually exclusive driver cascade, first-line treatment categories, survival endpoints, or multivariable models.*

**Supplementary Table 2. First-line targeted therapy generation among patients receiving first-line targeted therapy.**

| Targeted therapy generation group | Representative agent or regimen text | n | % of first-line targeted therapy group, N = 156 |
| --- | --- | --- | --- |
| EGFR third-generation therapy | Osimertinib-containing regimens | 76 | 48.7 |
| ALK later-generation therapy | Alectinib-containing regimens | 35 | 22.4 |
| EGFR first- or second-generation therapy | Erlotinib- or afatinib-containing regimens | 28 | 17.9 |
| ALK first-generation therapy | Crizotinib-containing regimens | 15 | 9.6 |
| Other named targeted agent | Dabrafenib plus trametinib | 2 | 1.3 |

*Percentages are calculated among patients classified as receiving first-line targeted therapy (N = 156). Generation was assigned from regimen text when the targeted agent was identifiable. This supplemental classification was used to describe practice patterns and did not alter the primary first-line treatment categories, driver-group definitions, survival endpoints, or multivariable models. Percentages may not sum to 100% because of rounding.*
